# Trauma and pregnancy: Number 10 – October 2023

**DOI:** 10.1055/s-0043-1777339

**Published:** 2023-12-06

**Authors:** Carlos Alberto Maganha, Marcelo Augusto Fontenelle Ribeiro, Rosiane Mattar, Mauricio Godinho, Renato Teixeira Souza, Elton Carlos Ferreira, Sara Toassa Gomes Solha, Fernanda Santos Grossi, Larissa Mariz de Oliveira Godinho

**Affiliations:** 1 Faculdade de Ciências Médicas de São José dos Campos, São José dos Campos, SP, Brazil; 2 University and Gulf Medical University, Division Chair of Trauma, Burns, Critical Care and Acute Care Surgery, Sheikh Shakhbout Medical City, Mayo Clinic Abu Dhabi, United Arab Emirates; 3 Escola Paulista de Medicina, Universidade Federal de São Paulo, São Paulo, SP, Brazil; 4 Departamento de Cirurgia e Anatomia, Faculdade de Medicina de Ribeirão Preto, Universidade de São Paulo, Ribeirão Preto, SP, Brazil; 5 Universidade Estadual de Campinas, Campinas, SP, Brazil; 6 Pontifícia Universidade Católica de Campinas, Campinas, São Paulo, Brazil; 7 Policlínicas Municipal de Sorocaba, Sorocaba, SP, Brazil; 8 Hospital Geral da Universidade de Caxias do Sul, Caxias do Sul, RS, Brazil; 9 Departamento de Ginecologia e Obstetrícia, Faculdade de Medicina de Ribeirão Preto, da Universidade de São Paulo, Ribeirão Preto, SP, Brazil

## Key points

- The incidence of trauma during pregnancy is 6-8% (severe forms of trauma: 3-6%).
- Of pregnant women who need hospitalization due to trauma, 60% progress to delivery.
- Pregnant women are 1.6 times more likely to die in a trauma situation.
- The anatomical and physiological alterations of pregnancy interfere with the repercussions and the approach to trauma.
- Domestic violence represents the most common trauma mechanism for pregnant women and triggers several obstetric complications. Ideally, it should be identified during antenatal care.
- The diagnosis of placental abruption in car accidents deserves special attention.
- Ultrasound in the trauma room enables action in trauma care, and as a quick mechanism, the necessary information about the fetus and pregnancy (fetal FAST).
- Most imaging exams required for good trauma care do not represent harm to the pregnancy.
- Antenatal care plays an important role in preventing trauma during pregnancy.
- The joint action of the trauma surgeon and the obstetrician is recommended in the care of traumatized pregnant women, especially in severe cases and in pregnant women over 20-24 weeks.

## Recommendations

- When caring for pregnant women who suffered severe trauma, especially after 20 weeks, the uterus must be moved to the left to decompress the inferior vena cava.
- Pregnant women victims of severe trauma must receive priority care in trauma centers with obstetric support. If unavailable, prioritize a referral to trauma centers.
- In view of the need to perform chest drainage in pregnant women over 20 weeks, this should be done in the 3 ^rd^ or 4 ^th^ intercostal space anterior to the mid-axillary line to avoid diaphragmatic elevation typical of pregnancy.
- Fluid and transfusion replacement – if needed – should be based on the shock index and favor blood transfusion over the massive use of crystalloids.
- In car accidents involving pregnant women, even if the abdomen is not reached, the diagnosis of placental abruption should be pursued.
- In car accidents, consider the observation with continuous fetal monitoring for a minimum period of six hours.
- Perimortem cesarean section should be indicated in pregnant women with a uterus above the umbilicus if there is no return to maternal spontaneous circulation after four minutes of cardiopulmonary resuscitation (CPR) or if it is not possible to estimate the duration of cardiopulmonary arrest (CPA).
- Screening for obstetric violence should occur systematically during the first antenatal visit and be repeated every trimester and at the postpartum visit.
- The obstetrician should advise about the correct use of the seat belt during antenatal care.

## Background

The incidence of trauma during pregnancy is around 6-8%, and when considering serious forms of trauma, it affects 3-6% of all pregnancies. 1 2 Three to four trauma occurrences in pregnant women per 1,000 will require hospitalization and 60% of these will progress to delivery. Severe trauma represents the main cause of non-obstetric maternal death in the world. 1 3 Compared to non-pregnant women, pregnant women are twice as likely to suffer severe trauma and 66% more likely to progress to death. 4 Pregnancy currently represents an isolated predictor of post-trauma mortality, and pregnant women are 1.6 times more likely to die than non-pregnant women. 3 5 Thus, knowledge of the particularities of this dyad and the specific aspects of pregnancy in trauma are of interest to obstetricians and trauma surgeons. 1 2

## Can pregnancy interfere with trauma care?

Several physiological alterations during the pregnancy-puerperal cycle may interfere in the care and management of the pregnant woman victim of trauma. These physiological alterations can simulate pathological conditions that are found in trauma and confuse the physician during care because of the potential to simulate normal situations. 2 5 6 Chart 1 summarizes these alterations with the respective possible interferences in the management of these traumas.

**Chart 1.** Physiological changes during pregnancy and possible implications for trauma care

Physiological changes	Clinical implications
Cardiovascular Blood volume ⇈ x erythrocyte mass ↑ Heart rate ↑ (15-20 bpm) ↓ Blood pressure (2 ^nd^ trimester) Plasma volume ↑ (30%-40%) ↓↓ Peripheral resistance ↑ Uterine volume (3 ^rd^ trimester) → cava compression (pregnant woman in supine position)	Physiological “anemia” (normal hemoglobin ≥ 11 g/dL) Difficulty in diagnosing hypovolemic shock ↓ Venous return when patient in supine position (↓ preload)
Pulmonary Elevation of the diaphragmatic dome by 4 cm Functional residual capacity ↓ 20% Inspiratory capacity ↑	Respiratory alkalosis Lower apnea time tolerance
Renal ↑ Glomerular filtration ↑ Plasma volume	↓ Creatinine, urea and plasma uric acid
Coagulation ↑ Factors I, VII, VIII and X and XII ↑ Fibrinogen ↑ Von Willebrand Factor Resistance to activated C protein	↑ Risk of thromboembolism
Gastrointestinal ↑ Gastrin ↓ Esophageal sphincter activity ↓ Gastrointestinal motility	↑ Production of gastric secretion ↑ Risk of bronchoaspiration

Source: Prepared by the Assistance Group for Trauma in Pregnant Women (CNEGAR-Febrasgo and SBAIT).

### Cardiovascular system

The cardiovascular system undergoes profound changes during the pregnancy-puerperal cycle, either by the influence of placental hormones and nitric oxide, the placentation process or even the anatomical alterations resulting from uterine growth, especially after 20 weeks. The main characteristics found in pregnancy are increased heart rate (increasing up to 32 weeks) and plasma volume, which determine the progressive increase in cardiac output during pregnancy. There is also a reduction in peripheral vascular resistance determining a decrease in arterial blood pressure, particularly in the second trimester of pregnancy. From 20 weeks onwards, the gravid uterus prevents normal venous return to the heart when the pregnant woman assumes the supine position, determining compression of the inferior vena cava. This situation is circumvented when the pregnant woman assumes the left lateral decubitus. In trauma care, especially in severe cases, this condition is very relevant, as it determines the need for alternative positions for adequate medical care, ideally the decubitus with an angle of 20° to the left or with manual decompression of the uterus to the left side. Special attention should be given to patients who may have spinal cord trauma compromising the thoracic and/or lumbar spine. In these cases, en bloc mobilization or manual displacement only of the uterus represent the appropriate maneuvers, avoiding trunk movement. 5

Another important context related to increased plasma volume is blood hemodilution. This condition occurs due to a much higher plasma increase than the erythrocyte volume during pregnancy. Thus, a condition of normality is observed in the face of lower hemoglobin and hematocrit concentrations than those usually found outside of pregnancy (“physiologic anemia” of pregnancy). Hemoglobin concentration decreases from 13 g/dL (usual situation outside pregnancy) to 11 g/dL in the first trimester and 10.5 g/dL in the second and third trimester of pregnancy. Faced with such repercussions, it is important to emphasize that more than laboratory alterations, in the face of trauma in pregnant women, we must use hemodynamic parameters. The quantification of diuresis is characterized as one of the main hemodynamic variables during the initial care of trauma victims. 4

### Respiratory system

Pregnant women undergo marked changes in their thoracic anatomy. These modifications involve both an increase in the anteroposterior and transverse diameters of the thorax by approximately 2 centimeters and a diaphragmatic elevation of up to 4 centimeters. Therefore, technical care at the time of drainage should be reinforced, such as digital exploration of the cavity before insertion of the chest drain, as this can contribute to reduce accidents during the procedure, such as, for example, the placement of the drain in the abdominal cavity. Depending on the uterine volume and elevation of the diaphragm, the higher insertion, above the 5 ^th^ intercostal space, will make the procedure safer. 7 Progesterone, by stimulating the respiratory center, plays an important role in increasing the current volume (or tidal volume). This change determines the condition of hyperventilation, which is important to facilitate gas exchange during pregnancy, although it leads to a condition of physiological respiratory alkalosis (with a decrease in pCO2 to less than 30 mmHg). This process is naturally compensated for by the excretion of bicarbonate (whose concentration decreases in the plasma), so that the pH does not change. 7 Table 1 summarizes the alterations found in the blood gas analysis of pregnant women.

**Table 1.** Blood gas changes found during pregnancy

	Non-pregnant	Pregnant
PO _2_ (mmHg)	75 to 100	105 ↑
PCO _2_ (mmHg)	37 to 40	27 to 32 ↓
Arterial pH	7.35 to 7.40	7.40 to 7.45 ↑
Bicarbonate (mmol/L)	22 to 26	18 to 21 ↓

Source: Adapted from Greco et al. (2019). 8

On the other hand, these changes, which are harmonically compensated under normal conditions, may influence pregnant women’s response to conditions of low oxygenation or apnea. Thus, they are less adaptable to longer periods of apnea, becoming more vulnerable to hypoxia. This aspect cannot be overlooked in trauma care during pregnancy.

### Renal system

With the increase in cardiac output and the decrease in peripheral resistance, there is a concomitant increase in renal plasma flow and glomerular filtration rate.

Plasma creatinine concentration reaches average values of 0.5-0.8 mg/dL, while uric acid concentration drops to levels below 3 mg/dL in the first trimester, rising slightly in the third trimester of pregnancy. 3 9

### Coagulation system

Important alterations occur in pregnant women’s blood clotting, leading to the condition of hypercoagulability. There is an increase in fibrinogen, which can reach 400-600 mg/dL, an increase in clotting factors (factors I, VII, VIII, X, XII) and a decrease in thrombolytic factors. Although these alterations are important to avoid greater blood loss in the physiological process of childbirth and puerperium, they determine a greater thromboembolic risk for pregnant and puerperal women, especially when associated with trauma mechanisms such as fractures, bleeding and/or resulting from long periods of immobilization.

### Gastrointestinal system

The gastrointestinal system (esophagus, stomach, gallbladder and intestine) remains atonic throughout pregnancy because of hormonal action, particularly progesterone, on the smooth muscles of these organs. This alteration is associated with the gradual growth of the gravid uterus, which determines an increase in abdominal pressure. In view of this situation, the relaxation of the gastroesophageal sphincter and the increase in intra-abdominal pressure favor situations of reflux and, in case of trauma care, a greater risk of aspiration of gastrointestinal contents. 6 This should be particularity monitored closely in the trauma care room, as well as in surgeries and intensive care units.

## What are the possible mechanisms of trauma in pregnant women?

The main mechanisms of trauma in pregnant women are: domestic violence, car accidents, falls, penetrating/perforating trauma, attempted murder, burns and inhalation of toxic substances and/or gases. 1 8 10 11

### Domestic violence

Domestic violence very likely represents the main cause of trauma to pregnant women. Approximately 4-8% of pregnant women suffer some type of violence during their pregnancy. 11 These numbers can be much higher if underreporting is taken into account. Most cases of domestic violence originate from intimate partner violence, and the resulting injuries are frequently found in the head and neck region, and also on the back. 2 The risks to pregnancy inherent to intimate partner violence go beyond the trauma and may mean an increase in intercurrences in the pregnancy-puerperal cycle, namely: fetal growth restriction, anemia, smoking, prematurity, stillbirth, premature rupture of membranes and placental abruption. 5 12 13 In the face of suspected domestic violence, it is important that the health professional and/or the health unit perform the compulsory notification to the police authority in accordance with Ordinance GM/MS No. 78, of January 18, 2021, 14 in line with Article 4 of Law number 10.778, of November 24, 2003. 15

### Car accidents

Car accidents represent a significant portion of trauma in pregnant women and are the most related to blunt injuries (55-70%). 16 Around 200-300 pregnant women/100,000 live births are involved in car accidents. However, more than 50% of them reported not having been properly instructed about the use of seat belts by their physicians. 2 10 16 Another important aspect mainly related to serious accidents is the use of drugs and alcohol, as approximately 45% of serious car accidents involving pregnant women are associated with alcohol use. 4 In this type of accident, direct blunt trauma to the fetus is uncommon, given the protective role played by the uterus and annexes to the fetus, thus, the worst consequence is the possibility of placental abruption, which is also the most feared mechanism of fetal compromise. 2 13 17 Placental abruption can occur in up to 40% of pregnant women that are seriously injured in car accidents. 2 Although placental abruption is more common in severe trauma, it can also occur in apparently mild trauma. 17 In severe trauma suffered by pregnant women over 35 weeks, the occurrence of splenic and hepatic injuries is more common, because of the greater congestion of these organs during the pregnancy period. 16

### Falls

Due to joint instability, pregnant women are more susceptible to falls. Although frequent, they do not usually lead to fetal risk, given the protection offered by the uterine cavity. It is believed that one in four pregnant women will have at least one fall during pregnancy. 18 19 Most falls occur at home, and falls from one’s own height are frequent. About 39% involve stairs. 20 Fractures of the extremities are common, most commonly involving the lower limbs. Unfavorable obstetric effects involving falls are premature birth (RR: 4.4), placental abruption (RR: 8), fetal distress (2.1), fetal hypoxia (2.9) and stillbirth (2.0). 12 21

### Burns

Injuries caused by burns have different mechanisms from those of other forms of trauma: direct thermal injury to the tissue, injury due to inhalation in the lungs, and accumulation of toxic substances in the bloodstream. The estimated incidence of burns is 0.17 per 100,000 pregnancies. The impact of the burn will depend on the depth and extent of the affected area. When the affected area exceeds 40% of the body surface, fetal mortality can reach 100%. 1 The mortality rate can also be influenced when the pregnant woman inhales smoke, as the inhalation of carbon monoxide freely crosses the placental barrier and avidly binds to fetal hemoglobin, triggering tissue hypoxia. 16 21

### Penetrating/perforating trauma

Penetrating/perforating trauma is mainly related to stab and firearm injuries. It is uncommon during pregnancy (2.3 per 100,000 live births) and associated with low rates of maternal mortality. 1 18 This characteristic is attributed to displacement of the abdominal viscera. However, perinatal mortality can reach 40% and results from prematurity or direct fetal injury. 1 21

## What is the role played by ultrasound in the trauma room?

Ultrasound in the trauma room in non-pregnant patients is a very important element in the surgeon’s decision-making, especially in assisting victims of abdominal and thoracic injuries with hemodynamic instability. The so-called FAST is a quick test that looks for the presence of fluid collected in the abdominal region, and more currently in the abdominal and thoracic regions (eFAST). 22

The eFAST consists of: 1) longitudinal view in the right upper quadrant for analysis of the liver, right kidney and Morrison’s pouch; 2) longitudinal view in the left upper quadrant to analyze the spleen, left kidney and splenorenal space; 3) transversal views in the suprapubic region for analysis of the bladder and uterine and rectouterine pouch (impaired analysis during pregnancy); 4) transverse subxiphoid view to investigate pericardial effusion and injuries in the left lobe of the liver; and 5) longitudinal views in the right and left thoracic apexes and bases to search for pneumothorax and/or presence of fluid. This exam should last between 3-5 minutes and is very accurate in detecting bleeding and free fluid in the cavities. 22 23

In the same logic of rapid evaluation during pregnancy, it is possible to use ultrasound in the trauma/emergency room for a primary fetal analysis (fetal FAST). Within the concept of multidisciplinary trauma care for pregnant women, it can be an important instrument in detecting fetal elements that are determinant for decision-making, even if it is not carried out by obstetricians. Like eFAST, fetal FAST must adhere to very objective fetal data: presence of fetal heartbeat, placental position, subjective assessment of amniotic fluid volume, and assessment of femur length. The measurement of the femur is the best isolated measurement for estimating gestational age in the third trimester (femur > 4 centimeters = viable fetus). The exam should not extend over time in search of complementary information, such as diagnosis of estimated due date, objective determination of amniotic fluid volume and assessment of fetal circulation, which should be secondary assessments to the initial care. 24 25

## What obstetric repercussions can we expect in the case of trauma?

The main obstetric complications for pregnant women in the case of trauma are: miscarriage, prematurity, placental abruption, rupture of ovular membranes, uterine rupture and fetal death. These complications vary according to gestational age, trauma mechanism, severity and, evidently, the degree of maternal instability in relation to the trauma. 12 Some deserve consideration.

### Placental abruption in pregnant women with trauma

Placental abruption is a serious obstetric condition due to the maternal, fetal and neonatal morbidity and mortality it entails. It occurs in the second half of pregnancy with an incidence of 2-10 cases/1,000 live births. 26 One of the causes of placental abruption is mechanical trauma. It is the most common complication in pregnant women victims of this type of trauma, and can occur even in less serious situations. Evidently, the increase in the severity of the trauma increases the percentage of occurrence of this pathology. 12 Placental abruption is present in 40% of the cases of car accidents with severe trauma to the pregnant woman. However, even in less severe abdominal trauma, we can find an association with placental abruption in 3% of cases. 26 Two main mechanisms are involved in the genesis of placental abruption in cases of car accidents: the increase in negative pressure imposed on the abdomen and placental traction/tension failure, which does not follow uterine movement. Due to these factors, shear forces cause cleavage of the decidual-placental interface and subsequent detachment of the placenta from its insertion bed. An important fact to be highlighted is that the detachment caused by the uterine movement can be delayed, which should be taken into account in the monitoring of fetal vitality in these situations. 6 12 The diagnosis of placental abruption is clinical. Acute, intense and non-rhythmic abdominal pain is the main clinical predictor of an unfavorable outcome. However, depending on the level of consciousness at which the pregnant woman is admitted, it will not be a plausible parameter for evaluation. Sudden bleeding of variable volume, increased uterine sensitivity, hypertonia, tachysystole, maternal hypotension and changes in fetal vitality culminating in fetal death may be present in the clinical picture. 26 Ultrasound has low sensitivity (25-60%) for identification of retroplacental hematoma in its initial phase. In the case of acute bleeding, an isoechogenic image is formed, with the placenta making it difficult to identify. In cases of placental abruption that culminate in fetal death, the degree of bleeding and the percentage of detached placental area are significant. In all cases of fetal death identified in the first steps of care to the trauma victim, severe placental abruption should be strongly suspected. In these cases, it is considered that > 50% of placental separation occurred, and 20% of cases are associated with disseminated intravascular coagulation. Massive bleeding can be hidden with little or no exteriorization via the vagina, which makes the clinical diagnosis of placental abruption more difficult. Hence the alert for fetal death as a sign of hemorrhagic severity in the context of trauma. The occurrence of Couvelaire’s uterus is also a constant concern in this scenario. Blood infiltration alters the activity of myometrial fibers, making the response to drugs to correct atony less effective. It presents a high risk of evolving, with the need for hysterectomy to contain the bleeding. After maternal stabilization, continuous cardiotocographic monitoring should be established for at least four to eight hours. It is an instrument for immediate detection of deterioration in fetal vitality, with a high possibility of having placental abruption in its genesis. The tachysystole pattern can also be a predictor of placental abruption. When ≥ 6 contractions/hour are recorded, the possibility of extending continuous fetal monitoring for up to 24 hours is suggested. 25 Laboratory tests should be requested to assess volume loss and the degree of shock: blood gas analysis, complete blood count, coagulogram, blood typing, lactate and the best predictor of coagulopathy, fibrinogen. However, note that at the beginning of the hemorrhagic condition, laboratory tests do not reliably reflect the acute blood loss (which may be hidden and massive in some cases). They are suitable for post-transfusion control. The level of fibrinogen has the best correlation with the severity of the shock. Fibrinogen levels < 200 mg/dL have a positive predictive value of 100% for identifying severe conditions. The expected goals during treatment with the patient are: hemoglobin > 8 g/dL, hematocrit from 21% to 24%, platelets > 50 thousand, fibrinogen > 200 mg/dL and TAP and APTT < 1.5 times the control values.

### Uterine rupture

Uterine rupture is a rare event associated with trauma (<1%). It may be secondary to perforations by pelvic bone fragments or related to direct trauma in major accidents, especially in the third trimester. Although uncommon and given the pelvic congestion, it is usually severe. 12

## Which imaging exams can and should be performed in the care of pregnant victims of trauma?

Most imaging exams indicated for adequate trauma care in pregnant women are not associated with greater risk and can be performed when necessary. The acceptable level of fetal irradiation, particularly in the first trimester, is up to 50 mGy or 5 rad. 27 28 29 The vast majority of tests used in the management of pregnant women with trauma have fetal radiation below the maximum level, as demonstrated in table 2 . 27

**Table 2.** Fetal radiation doses in common exams in the trauma room

Exam	Fetal dose (mGy)
Head or neck CT scan	0.001-0.01
Extremity x-ray	< 0.001
2-view chest x-ray	0.0005-0.01
Abdominal or pelvic X-ray	0.1-3.0
Chest tomography	0.1-0.66
Lumbosacral spine X-ray	1-10
Abdominal tomography	1.3-35
Pelvic tomography	10-50

Source: Adapted from Committee Opinion No. 723 (2017). 27

During admission to the trauma room, imaging tests should be objective in order to identify potentially lethal injuries. 28 Among them, the following are performed in a protocol manner:

1. Anteroposterior chest X-ray (assess fractures, pneumothorax or hemothorax, diaphragmatic hernia);
2. Anteroposterior pelvic X-ray (assess fractures and pelvic instability);
3. e-FAST (assess the presence of pneumothorax and free fluid in the pericardial sac and abdominal cavity).

Tomography represents the gold standard examination for identifying central nervous system and spinal cord injuries, and for investigating intracavitary bleeding, and it contributes to surgical planning. However, the patient’s hemodynamic stability is essential to perform such an examination. Pelvic and/or abdominal tomography should not be avoided due to pregnancy, even under the assumption that the radiation reaches the fetus, in view of its importance for detecting visceral injuries. 28 29

X-ray exams must be requested so that musculoskeletal injuries can be identified and treated. As long as the team is trained and there is possibility, the fetal FAST is performed for guidance on fetal viability and vitality. Magnetic resonance imaging in pregnant women has no complications for the fetus. Gadolinium should not be used as a contrast method, as it is associated with inflammatory processes and stillbirth. In scenarios of stability and low suspicion of multiple injuries, MRI may represent a reasonable substitute for tomography, in view of its quality of soft tissue images and fetal non-irradiation. 27

## What obstetric approaches are necessary for pregnant trauma victims?

### Assessment of fetal vitality

Note that the focus of care for pregnant women victims of trauma is the maintenance of maternal life. Fetal evaluation measures should be taken particularly after maternal stabilization and/or in extreme cases, such as perimortem cesarean section in the case of CRA. 11 The main method described for assessing fetal vitality in traumatized pregnant women, especially after 24-26 weeks, is continuous antepartum cardiotocography. This method is particularly important in the case of major car trauma, where the chance of developing placental abruption is greater. It should be started as soon as maternal conditions allow and performed at least six hours after admission (as long as the pregnant woman has not associated bleeding and/or uterine contractions). The safe time of this assessment is discussed. Some services may suggest a follow-up of up to 24 hours, particularly in cases of suspected placental abruption or genital leakage (fluid or blood). However, most recommendations do not exceed eight hours of maintenance of this monitoring. 30 31 Since most severe traumas are attended by trauma centers without maternity units and a neonatal intensive care unit, the transfer of these pregnant women to greater centers with this complexity should be recommended as soon as maternal conditions allow. 30

### Medicines

The use of corticosteroids and magnesium sulfate should be considered when premature birth is a possibility, as it will bring benefits in terms of morbidity and mortality of children born prematurely to these mothers. However, an important alert: childbirth, especially when necessary due to a serious maternal condition, should not be postponed to perform these therapies. 32

### Perimortem cesarean section

Perimortem cesarean section is considered a resuscitation maneuver in pregnant women. It is indicated if there is no return to maternal spontaneous circulation after four minutes of CPR or if it is not possible to estimate the patient’s CPA time. 33 In all cases, it should only be performed if the uterine fundus extends above the umbilical scar, aligning the possibility of fetal viability and that the uterine fundus at this time represents an important factor of compression to the inferior vena cava, interfering with maternal venous return. 34 After the procedure, with uterine emptying, there is an increase in pre-cardiac load, and maternal blood flow is more easily reestablished, favoring the return of spontaneous circulation and the reduction of CPA time. A review of cases that included 38 patients showed that 12 out of 20 pregnant women duly monitored showed return of spontaneous circulation soon after delivery and, of these, 30 resulted in a viable newborn after delivery. 34 The early performance of perimortem cesarean section facilitates resuscitation efforts and decreases the risk of fetal anoxia. However, it is important to emphasize that fetal viability or vitality does not influence the indication of the procedure. Thus, fetal monitoring is not recommended during care.

### Prophylaxis of RH isoimmunization

Fetomaternal hemorrhage occurs in 10-30% of pregnant trauma patients. Rh-negative pregnant women should receive anti-D immunoglobulin (Rh-D) in the condition of trauma with risk of maternal-fetal blood exchange. The appropriate dose of anti-D immunoglobulin depends on the amount of exposure. A standard dose of 300 μg anti-D immunoglobulin will protect up to 30 mL of fetal blood, but abdominal trauma can often exceed 30 mL of fetal blood in maternal circulation. Therefore, blunt abdominal trauma is more likely to require more than one dose of anti-D immunoglobulin. 31 35 The administration of anti-D immunoglobulin is recommended within 72 hours to avoid future anti-Rh sensitization. When this is not possible, it is still recommended to perform it in longer terms, even incurring less effectiveness. 35

## What is the role played by the obstetrician in preventing trauma during pregnancy?

### Antenatal approach to domestic violence

Antenatal care can play a fundamental role in creating awareness about the signs, types and degrees of domestic violence and in order that the signs of violence, even incipient, are perceived. Creating a safe environment in which women can understand, recognize and report violence is fundamental to fight it. Furthermore, it is a way to offer proper multidisciplinary care to those who have suffered violence. 36 The training of professionals to recognize, offer embracement and respond to violence is fundamental for the implementation of screening for these occurrences during antenatal care. Screening should occur periodically; during the first antenatal visit, and repeated every quarter and at the postpartum visit. 37 There is no consensus on which is the best approach among the different options described in the literature, but it should be one that: 1) promotes preventive actions with educational interventions; 2) promotes channels of communication with the patient, so that she feels safe and embraced; 3) involves a multidisciplinary team; and 4) results in good adherence by the team, which can identify the different types of violence (physical, psychological, sexual, patrimonial and moral). During the service, the team must be prepared to deal with the spontaneous report of domestic violence or with the performance of direct and indirect questions for risk assessment. Chart 2 shows suggestions for a direct and indirect approach to screening for violence against women. 37 In addition to a direct or indirect approach, it is important to be aware of signs that lead to the suspicion of violence, such as the presence of the following: chronic, vague and repetitive disorders; late entry into antenatal care; very controlling companion; recurrent urinary tract infection (no secondary cause found); chronic pelvic pain; irritable bowel syndrome; disorders in sexuality; complications in previous pregnancies, recurrent miscarriages; depression; anxiety; history of suicide attempt; physical injuries that are not adequately explained. 38

**Chart 2.** Suggestions for screening for violence against women

**Direct questions**
• As you may know, nowadays it is not uncommon to hear about people who have been physically, psychologically or sexually assaulted throughout their lives, and we know that this can affect their health, even years later. Has this ever happened to you?
• I have seen problems like yours in people who are physically abused. Did this happen to you?
• Does someone hit you?
• Have you ever been forced to have sex with someone?
**Indirect questions**
• Is everything okay at home with your partner?
• Are you having problems with family relationships?
• Do you feel humiliated or attacked?
• Do you think problems at home are affecting your health?
• Do you and your husband (or son or father or relative) fight a lot?
• When you argue, does he get aggressive?

### Antenatal approach to falls and car accidents

**Figure 1.**
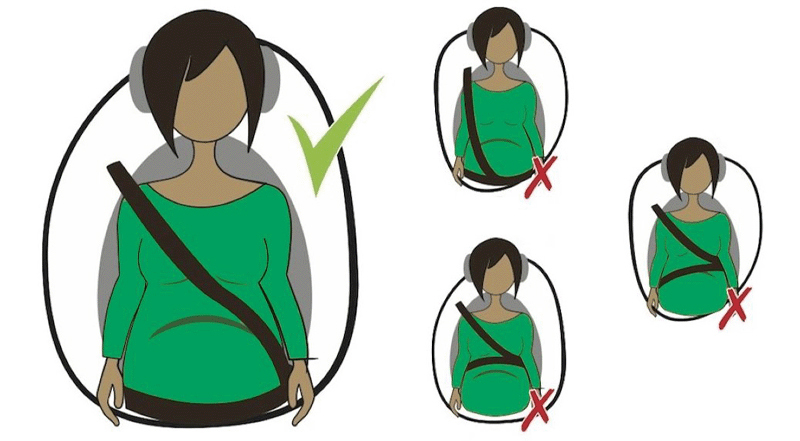
Correct seat belt use during pregnancy Source: https://bestcarseathub.com/blog/wearing-a-seat-belt-during-pregnancy-how-you-may-be-doing-it-wrong/

The obstetrician’s preventive action in the event of trauma during pregnancy mainly refers to educational aspects about behaviors that may put pregnant women and the fetus at risk. 17 The hormones produced during pregnancy act on the joints, increasing ligament laxity; fetal growth and weight gain can cause changes in body dynamics and balance axis; the action of progesterone on the central nervous system can decrease the level of attention. These physiological changes during pregnancy predispose to the occurrence of accidents and should be discussed during antenatal care. As mentioned, the hormonal action on the joints, associated with uterine growth and the change in the pregnant woman’s center of gravity leads to greater exposure to falls and eventual fractures of the extremities. Thus, it is very important that the antenatal care professional recommends the use of more comfortable and stable shoes during pregnancy, as well as domestic care with carpets and safety on stairs. 6 Although car accidents are an important cause of trauma during pregnancy, the need for proper use of seat belts is little discussed during antenatal care. Many pregnant women even stop using it due to discomfort or fear of hurting the fetus. Less than 50% of pregnant women who had accidents and were questioned about use of the seat belt reported having received guidance during antenatal care. When they use it, a significant portion of women does it inadequately. 3 6 21 The use of seat belts should always be advised, even if the pregnant woman is not driving the vehicle. The pelvic part of the belt should fit under the abdomen and across the upper thighs, and the thoracic part should cross the middle of the shoulder, passing between the breasts and lateral to the abdomen. None of the straps should pass over the gravid uterus ( Figure 1 ), or even be placed behind the chest, arm or pelvis, under penalty of compromising the safety of the mother and fetus. In vehicles equipped with an airbag, the seat must be moved as far as possible to allow safe contact with the steering wheel and pedals. An important recommendation in car accidents regards the role of the airbag. There are no studies demonstrating that its opening can trigger traumatic uterine injuries, and it constitutes a fundamental element in the prevention of severe brain trauma. Therefore, the attitude of deactivating this vehicle safety device should be strictly discouraged. 3 Regarding the use of bicycles and motorcycles, information should be given about the greater risk of falls due to modifications of the body axis and that direct impacts on the abdomen in an eventual fall can cause complications to the fetus.

Cell phone use while driving has been identified as an important cause of car accidents and seems to play a similar role to the use of alcohol in the loss of necessary attention.

## Final considerations

Trauma during pregnancy is an important topic for both the trauma surgeon and the obstetrician due to the great reciprocal influence. Its incidence is between 6% and 8% of all pregnancies and represents the main reason for maternal death from non-obstetric causes. Pregnancy is seen as an isolated predictor of post-trauma death, as pregnant women are twice as likely to suffer severe trauma and 1.6 times as likely to die. In addition, pregnant women who suffer severe trauma have a 60% chance of progressing to childbirth. The definition of referral is also important. It is recommended that pregnant women who suffer severe trauma are ideally referred to a trauma center with an integrated maternity. If unavailable, care for the mother should be the priority. During the care of pregnant women victims of severe trauma, no exam that is necessary for an adequate evaluation is contraindicated and ultrasound plays an important role in the trauma room both in the evaluation of traumatic injuries and in rapid fetal evaluation. The main motivating mechanism of trauma in pregnant women is domestic violence. It is believed that 4-8% of pregnant women experience domestic violence. The obstetrician plays a fundamental role during antenatal care in detecting these cases, since violence often occurs in the intimacy of the victim’s home. In these situations, an active search is recommended. Antenatal care is also equally important as an opportunity to provide guidance on the need to use the seat belt and safe shoes. Pregnancy is a great challenge in the face of trauma, given the presence of anatomical and physiological changes typical of this period, as well as the very presence of the maternal-fetal dyad that can substantially interfere with adequate medical care.

National Specialized Commission on High-Risk Pregnancy of the Brazilian Federation of Gynecology and Obstetrics Associations

President:

Rosiane Mattar

Vice-president:

Alberto Carlos Moreno Zaconeta

Secretary:

Mylene Martins Lavado

Members:

Maria Rita de Figueiredo Lemos Bortolotto

Fernanda Santos Grossi

Vera Therezinha Medeiros Borges

Inessa Beraldo de Andrade Bonomi

Janete Vettorazzi

Carlos Alberto Maganha

Renato Teixeira Souza

Felipe Favorette Campanharo

Sara Toassa Gomes Solha

Arlley Cleverson Belo da Silva

Elton Carlos Ferreira

Brazilian Society of Integrated Trauma Care (SBAIT)

President:

Mauricio Godinho (SP)

1st vice-president:

Amauri Clemente da Rocha (AL)

2nd vice-president:

Rogério Schneider (RS)

General secretary:

José Gustavo Parreira (SP)

1st secretary:

Fábio Henrique de Carvalho (PR)

2nd secretary:

Paulo Silveira (RJ)
